# Comment on: NETs in the spotlight: exploring NETosis markers for tracking disease activity in IgA vasculitis: Reply

**DOI:** 10.1093/rheumatology/keaf402

**Published:** 2025-07-26

**Authors:** Vafa Guliyeva, Fatma Gül Demirkan, Erdem Bektaş, Rabia Deniz, Zeliha Emrence, Özlem Akgün, Selen Duygu Arık, Ayşenur Doğru, Ayşe Tanatar, Neslihan Abacı, Sema Sırma Ekmekci, Ahmet Gül, Nuray Aktay Ayaz

**Affiliations:** Department of Pediatric Rheumatology, Istanbul University Istanbul Faculty of Medicine, Istanbul, Turkey; Department of Pediatric Rheumatology, Istanbul University Istanbul Faculty of Medicine, Istanbul, Turkey; Department of Internal Medicine, Istanbul Faculty of Medicine, Istanbul University, Istanbul, Turkey; Department of Rheumatology, Sakura City Hospital, Turkey, Başakşehir Çam and, İstanbul; Department of Genetics, Istanbul University, Aziz Sancar Institute of Experimental Medicine, Istanbul, Turkey; Department of Pediatric Rheumatology, Istanbul University Istanbul Faculty of Medicine, Istanbul, Turkey; Department of Pediatric Rheumatology, Istanbul University Istanbul Faculty of Medicine, Istanbul, Turkey; Department of Pediatric Rheumatology, Istanbul University Istanbul Faculty of Medicine, Istanbul, Turkey; Department of Pediatric Rheumatology, Istanbul University Istanbul Faculty of Medicine, Istanbul, Turkey; Department of Genetics, Istanbul University, Aziz Sancar Institute of Experimental Medicine, Istanbul, Turkey; Department of Genetics, Istanbul University, Aziz Sancar Institute of Experimental Medicine, Istanbul, Turkey; Istanbul Faculty of Medicine, Division of Rheumatology, Department of Internal Medicine, Istanbul University, Istanbul, Turkey; Department of Pediatric Rheumatology, Istanbul University Istanbul Faculty of Medicine, Istanbul, Turkey

Dear Editor, We thank the correspondent for their commentary [1] on our NETosis biomarkers study in pediatric IgA vasculitis (IgAV) [2].

The correspondent notes that Wang *et al.* [3] found elevated serum citH3 in adult IgAN patients, contrasting with our findings. Although our cohort lacked overt IgAN, 9 patients developed transient proteinuria, suggesting pediatric IgAV follows a milder, self-limiting course.

Our data showed elevated urinary citH3 (both active and inactive phases) despite normal serum levels. This compartmentalization aligns with other vasculitic conditions where tissue citH3 accumulation correlates with disease activity [4, 5], while serum levels may not reflect local inflammation. As clinicians, the discordantly low serum citH3 levels compared with other studies drew our attention, and we believe sharing this observation will be important as a preliminary insight for future research.

The correspondent’s hypothesis regarding urinary anti-endothelial cell antibodies releasing citH3 in pediatric IgAV is intriguing and may represent localized inflammation distinct from systemic NETosis in established nephritis. We also proposed the hypothesis of local accumulation in our study and recommended that future studies should examine serum, urine and tissue levels simultaneously, along with longitudinal assessment of children progressing to IgAN to help elucidate these mechanisms.

Regarding the correspondent’s additional questions: The two children requiring mycophenolate mofetil had persistent severe gastrointestinal symptoms and joint involvement that were refractory to steroid therapy, rather than nephrotic-range proteinuria or established IgAN. Interestingly, we observed that children with transient proteinuria did show numerically higher urinary citH3 levels compared with those without proteinuria, though this difference did not reach statistical significance in our small subgroup analysis. This observation warrants investigation in larger cohorts.

## Data Availability

The data underlying this article will be shared on reasonable request to the corresponding author.
